# Loss of PACS-2 delays regeneration in DSS-induced colitis but does not affect the *Apc*^Min^ model of colorectal cancer

**DOI:** 10.18632/oncotarget.22661

**Published:** 2017-11-26

**Authors:** Sarah L. Dombernowsky, Jeanette Schwarz, Jacob Samsøe-Petersen, Reidar Albrechtsen, Kim B. Jensen, Gary Thomas, Marie Kveiborg

**Affiliations:** ^1^ Biotech Research and Innovation Centre, University of Copenhagen, Copenhagen, Denmark; ^2^ Novo Nordisk Foundation Center for Stem Cell Biology, University of Copenhagen, Copenhagen, Denmark; ^3^ Department of Microbiology and Molecular Genetics, University of Pittsburgh School of Medicine, Pittsburgh, PA, USA

**Keywords:** DSS-induced colitis, colon cancer, Apc^Min^ model, PACS-2, ADAM17

## Abstract

PACS-2 is a multifunctional sorting protein that mediates cell homeostasis. We recently identified PACS-2 in a functional genome-wide siRNA screen for novel regulators of the metalloproteinase ADAM17, the main sheddase for ligands of the ErbB receptor family. Of note, we showed that *Pacs2*^-/-^ mice have significantly reduced EGFR activity and proliferative index in the intestinal epithelium. As EGFR signaling is highly mitogenic for intestinal epithelial stem cells, and plays essential roles in intestinal epithelial regeneration and tumor development, we have now examined the role of PACS-2 in these processes. Specifically, we analyzed the role of *Pacs*2-deficiency in a DSS-induced colitis model as well as in the genetic *Apc*^*Min*^ colon cancer model. We now report that loss of PACS-2 delays tissue regeneration after colonic injury with little effect on key inflammatory parameters. We did however not observe any apparent effects on tumor formation driven by excessive proliferative signaling downstream from APC-deficiency. Our findings reveal that the role of PACS-2 in regulating ADAM17-mediated shedding is not an obligate requirement for the epithelium to respond to the strong inflammatory or tumorigenic inducers in the models assessed here.

## INTRODUCTION

Phosphofurin acidic cluster sorting protein 2 (PACS-2) is a multifunctional sorting protein involved in trafficking of various cargo molecules. PACS-2 frequently acts in a complimentary manner to its close relative PACS-1, and the PACS protein family is central to ensuring the correct sorting and localization of a multitude of functionally diverse client proteins, with numerous downstream biological effects [1, 2]. We recently identified PACS-2 in a functional genome-wide siRNA screen for novel regulators of the transmembrane protease, a disintegrin and metalloproteinase-17 (ADAM17) [3]. We demonstrated that PACS-2 knockdown decreased the recycling of internalized ADAM17 protein, with a consequent reduction in the amount of active ADAM17 on the cell surface [3].

The dynamics of cell-surface localized ADAM17 are of importance because it cleaves a variety of cell-surface molecules, a process termed ectodomain shedding. Notably, ADAM17 sheds ErbB ligands, leading to activation of ErbB family receptors (epidermal growth factor receptor (EGFR), ErbB2, ErbB3, ErbB4) to trigger cell growth, survival, and migration [4]. ADAM17 is thus an important player in the regulation of epithelial development, growth and tissue homeostasis, and its deregulation has been implicated in many human diseases [5]. By decreasing cell-surface levels of ADAM17, PACS-2 knockdown led to a significant reduction in shedding of EGFR ligands. *In vivo*, this translated into reduced EGFR activition and proliferative index in the small intestinal epithelium and to a lesser extent in the colon of *Pacs2*^-/-^ mice [3]. By controlling ADAM17 cell-surface availability, PACS-2 thus appears to fine-tune ADAM17 and ErbB activity, with potential functional effects on intestinal homeostasis.

Intestinal epithelial homeostasis relies on stem cells in the mucosal crypts, which continuously replenish cells lost from the apex of villi [6]. EGFR signaling is highly mitogenic for intestinal stem cells [7] and therefore central for intestinal homeostasis and regeneration. Importantly, ADAM17-mediated EGFR activation is essential for gut regeneration, and *Adam17* and *Egfr*-deficient mice are more susceptible to colitis induced by oral administration of the sulfated polysaccharide dextran sodium sulfate (DSS) [8-11]. Ingestion of DSS ablates the colonic epithelium, resulting in inflammation and subsequent repair of the damaged areas [12]. While the precise mechanisms governing ADAM17- and EGFR-mediated protection against DSS-induced colitis remain unknown, the ErbB ligands and known ADAM17 substrates amphiregulin (AREG), epiregulin (EREG), and transforming growth factor (TGF)-α appear to be key effectors [8, 9, 13-15].

We hypothesized that as a regulator of ADAM17 and EGFR activities, loss of PACS-2 would influence intestinal epithelial pathologies. To this end, we treated *Pacs2*^-/-^ mice and wildtype littermates with DSS and evaluated disease progression and effects on intestinal histology. Another process that relies on EGFR signaling is the development of intestinal tumors [16], which originate from crypt stem cells [17]. Accordingly, we investigated the effects of PACS-2 loss in the *Apc*^Min^ mouse model of colorectal cancer. *Apc*^Min^ mice mimic familial colorectal cancer in patients with only one functional adenomatous polyposis coli (APC) gene allele, and APC is furthermore mutated in most sporadic human colorectal cancers [18]. As in humans, loss of heterozygosity of the remaining wildtype *Apc* allele results in the formation of multiple adenomas, and the *Apc*^Min/+^ mouse is therefore a commonly used model of colorectal cancer [19].

## RESULTS

### *Pacs2*^*-/-*^ mice show no significant changes in susceptibility to DSS-induced colitis

**Figure 1:**
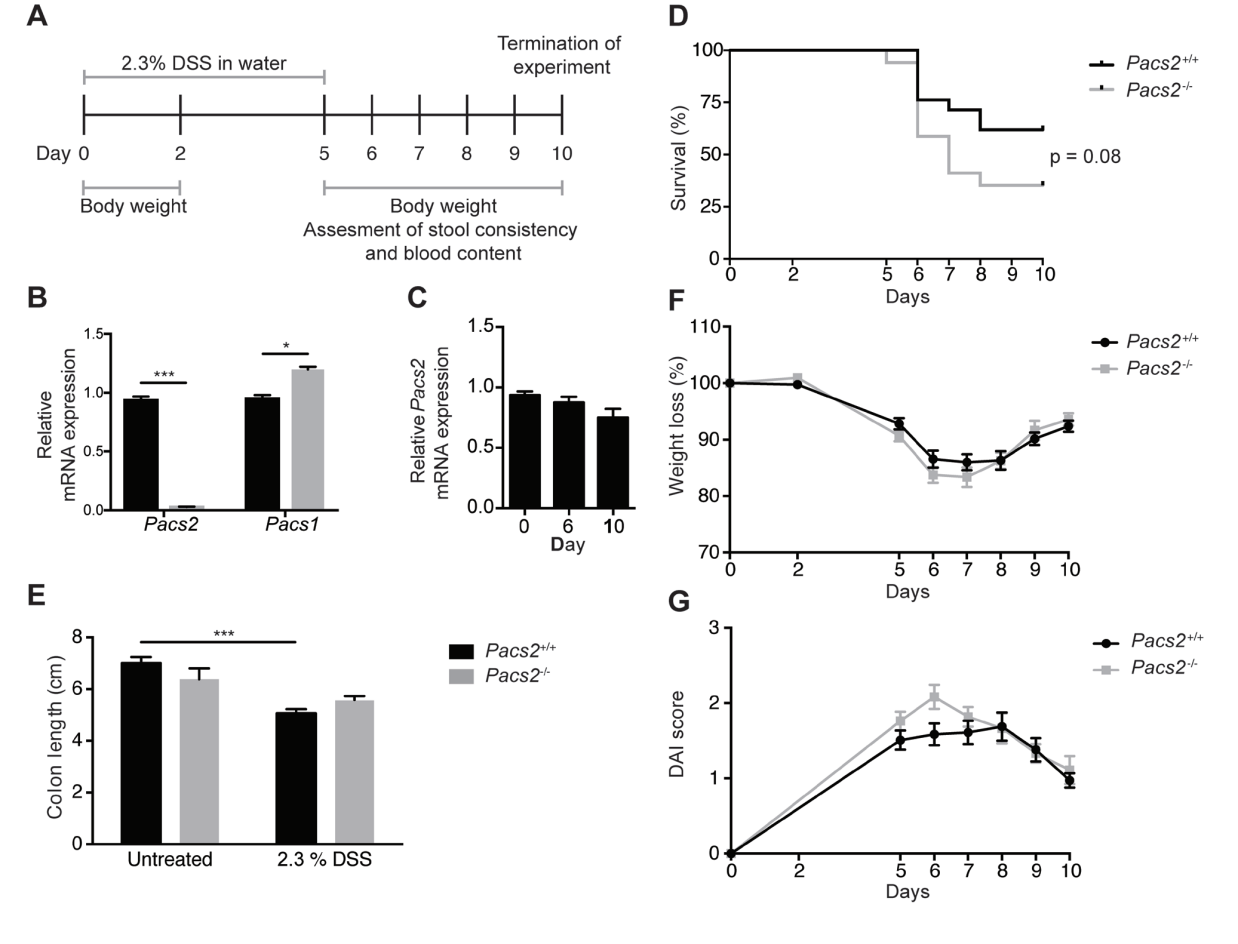
*Pacs2*^*-/-*^ mice show no significant changes in susceptibility to DSS-induced colitis **A.** Schematic of the DSS treatment regimen and data collection. Littermate *Pacs2*^+/+^ (control, *n* = 21) and *Pacs2*^-/-^ (*n* = 17) mice received 2.3% DSS in their drinking water for five days, followed by five days on regular water. Mice were weighed on days 0, 2, and 5-10, and stool consistency and blood content additionally monitored from days 5-10. **B.** Quantitative real-time PCR analysis of *Pacs2* and *Pacs1* mRNA levels in the colon of control (*n* = 5) and *Pacs2*^-/-^ mice (*n* = 4). *Gapdh* served as a control for normalization. **C.** Quantitative real-time PCR analysis of *Pacs2* mRNA levels in colonic tissue isolated from *Pacs2*^+/+^ mice at Day 0, 6 and 10 during DSS-induced colitis. **D.** Kaplan-Meier plot depicting the percentage survival of control (61 %) *versus Pacs2*^-/-^ (35 %) mice. Survival was evaluated by log-rank (Mantel-Cox) test (control, *n* = 21; *Pacs2*^-/-^, *n* = 17). **E.** Changes in colon length in control and *Pacs2*^-/-^ mice on day 6 of the DSS protocol (control, *n* = 9; *Pacs2*^-/-^, *n* = 4). **F.** Daily changes in body weight during DSS-induced colitis. Changes in body weight percentage were calculated by normalizing body weight at the specific day to the body weight at day 0 (control, *n* = 21; *Pacs2*^-/-^, *n* = 17). **G.** The Disease Activity Index (DAI) was calculated as the average of the weight loss score, stool score, and bleeding scores (control, *n* = 21; *Pacs2*^-/-^, *n* = 17). All data were pooled from three independent experiments. Graphs represent the mean ± S.E.M; ***p < 0.005 analyzed by two-way ANOVA.

To evaluate the effects of PACS-2 on intestinal inflammation and regeneration, groups of male *Pacs2*^-/-^ and littermate *Pacs2*^+/+^ (control) mice were administered 2.3% DSS in the drinking water, followed by 5 days on regular water (Figure 1A). Quantitative RT-PCR of mRNA isolated from colonic tissue of control and *Pacs2*^-/-^ mice verified the *Pacs2*-deficiency of *Pacs2*^*-/-*^ mice and importantly, revealed a small but statistically significant increase in the mRNA expression of PACS-1, the other member of the PACS protein family (Figure 1B). Of note, *Pacs2* mRNA expression was not significantly altered during DSS-induced colitis (Figure 1C). During the course of colitis, *Pacs2*^*-/-*^ mice tended towards poorer survival than littermate controls (35 % *vs*. 61 % survival at day 10, respectively; Figure 1D); however, this difference was not statistically significant (p = 0.08). Colon shortening, a macroscopic parameter of colitis severity, was observed to a similar extend in *Pacs2*^*-/-*^ and control mice (Figure 1E). Additionally, we found no significant difference in weight loss in *Pacs2*^-/-^ mice as compared to controls (Figure 1F).

To assess disease severity in more detail, we calculated the Disease Activity Index (DAI), adapted from the method first described by Cooper et al. [12]. The DAI is the average of three independent scores describing weight loss, stool consistency and stool blood content. The scoring system employed is outlined in Table 1. Although computation of the DAI showed a slightly increased disease severity in *Pacs2*^-/-^ mice as compared to controls, the change was not statistically significant (Figure 1G). Thus, while there was a tendency toward worse disease progression in *Pacs2*^-/-^ mice undergoing DSS-induced colitis, these changes were not significant.

**Table 1:** Scoring system used for calculation of Disease Activity Index

Score	Weight loss (%)	Stool consistency	Blood in stools
0	≤ 1	Normal	Negative Hemoccult test
1	> 1 - ≤ 5	Soft but formed	Positive Hemoccult test
2	> 5 - ≤ 10	Soft	Visibly bloody stool
3	> 10 - ≤ 20	Diarrhea	Rectal bleeding
4	> 20	-	-

Disease Activity Index = Average of the sum of the three scores. The scoring system is adapted from that described by Cooper et al. [12].

### *Pacs2*^*-/-*^ mice show comparable inflammation and tissue damage during DSS-induced colitis, but a slight delay in regeneration

**Figure 2:**
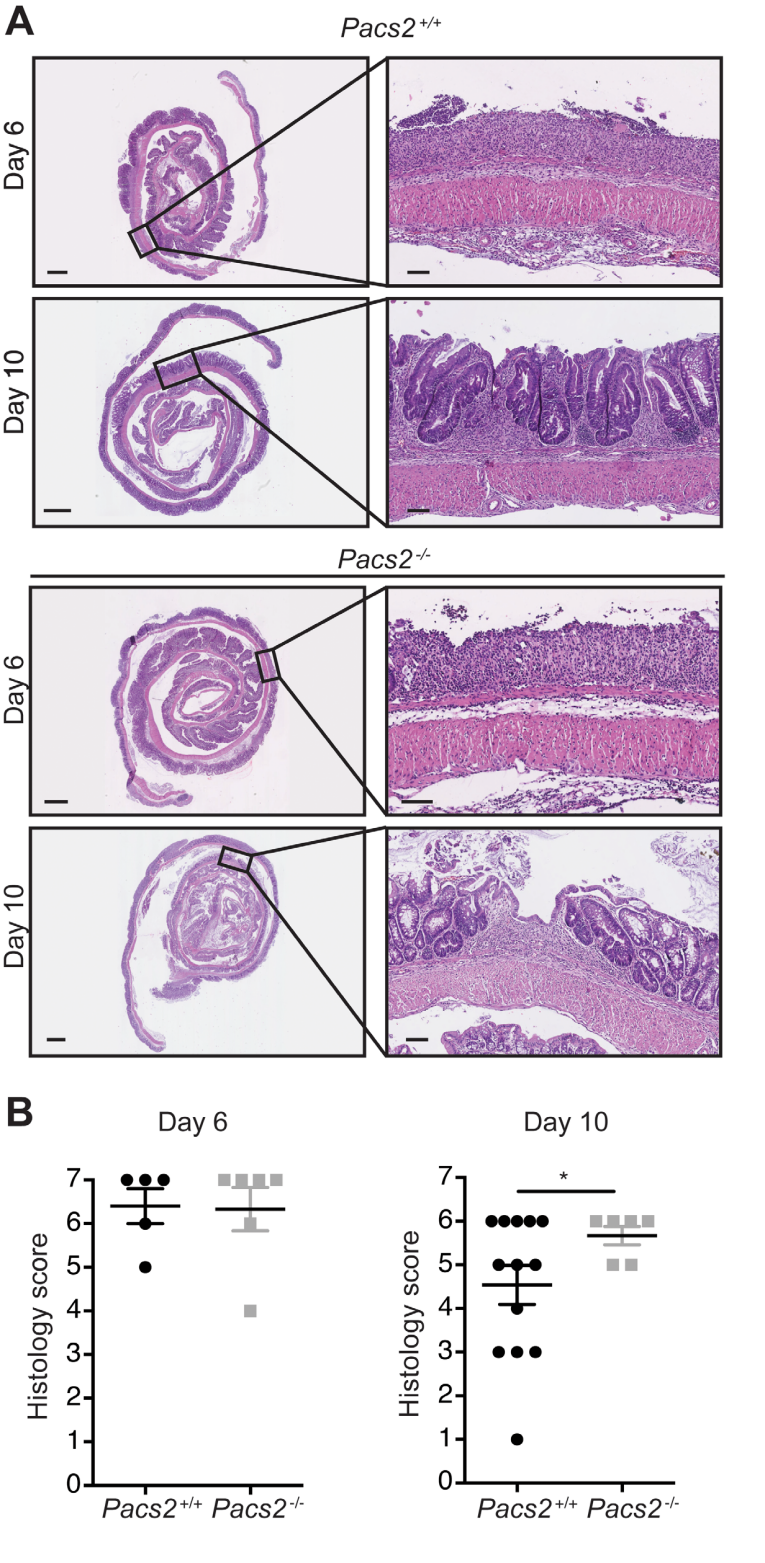
*Pacs2*^*-/-*^ mice show comparable inflammation and tissue damage during DSS-induced colitis Histological sections were scored blindly using the criteria outlined in Table 2. **A.** Representative images of colonic specimens from control and *Pacs2*^-/-^ mice isolated at day 6 and day 10. Scale bar overview pictures = 1 mm. Scale bar detail figures = 0.1 mm. **B.** Dot plots show the histology score, calculated as the sum of the epithelial damage score and the inflammation score. The individual scores were calculated as described in Table 2. All data represent the mean ± S.E.M. and were analyzed by unpaired Student’s t-test (*n* = 6-13 as indicated). Data were pooled from three independent experiments.

Histological examination revealed severe tissue damage with numerous mononuclear cells infiltrating the lamina propria of colons on day 6 of the DSS-induced colitis model in both control and *Pacs2*^-/-^ mice (Figure 2A). Day 6 was the time of most severe tissue damage, with some tissue regeneration being seen in histological sections from day 10 (Figure 2A). Tissue damage was quantified by a method adapted from Obermeier et al. [20]. Histological sections were scored blindly using the criteria outlined in Table 2 and a histology score was calculated by combining the epithelial damage score and the inflammation score. Consistent with the hematoxylin and eosin (H&E) images presented, there was no difference in the histology score between groups on day 6 of DSS-induced colitis (Figure 2B, left graph). On day 10, the histology score was significantly higher in *Pacs2*^-/-^ mice as compared to control mice (Figure 2B, right graph), which indicates a slight delay in tissue regeneration.

**Table 2:** Scoring system used for calculation of histology score

Score	Epithelial Damage (E)	Inflammation (I)
0	Normal morphology	Normal morphology
1	Loss of goblet cells	Mild inflammation
2	Loss of goblet cells in large areas/smaller areas with loss of crypts	Moderate inflammation
3	Moderate loss of crypts	Severe inflammation
4	Loss of crypts in large areas	-

Histology score = E+I. The scoring system is adapted from that described by Obermeier et al. [20].

**Figure 3:**
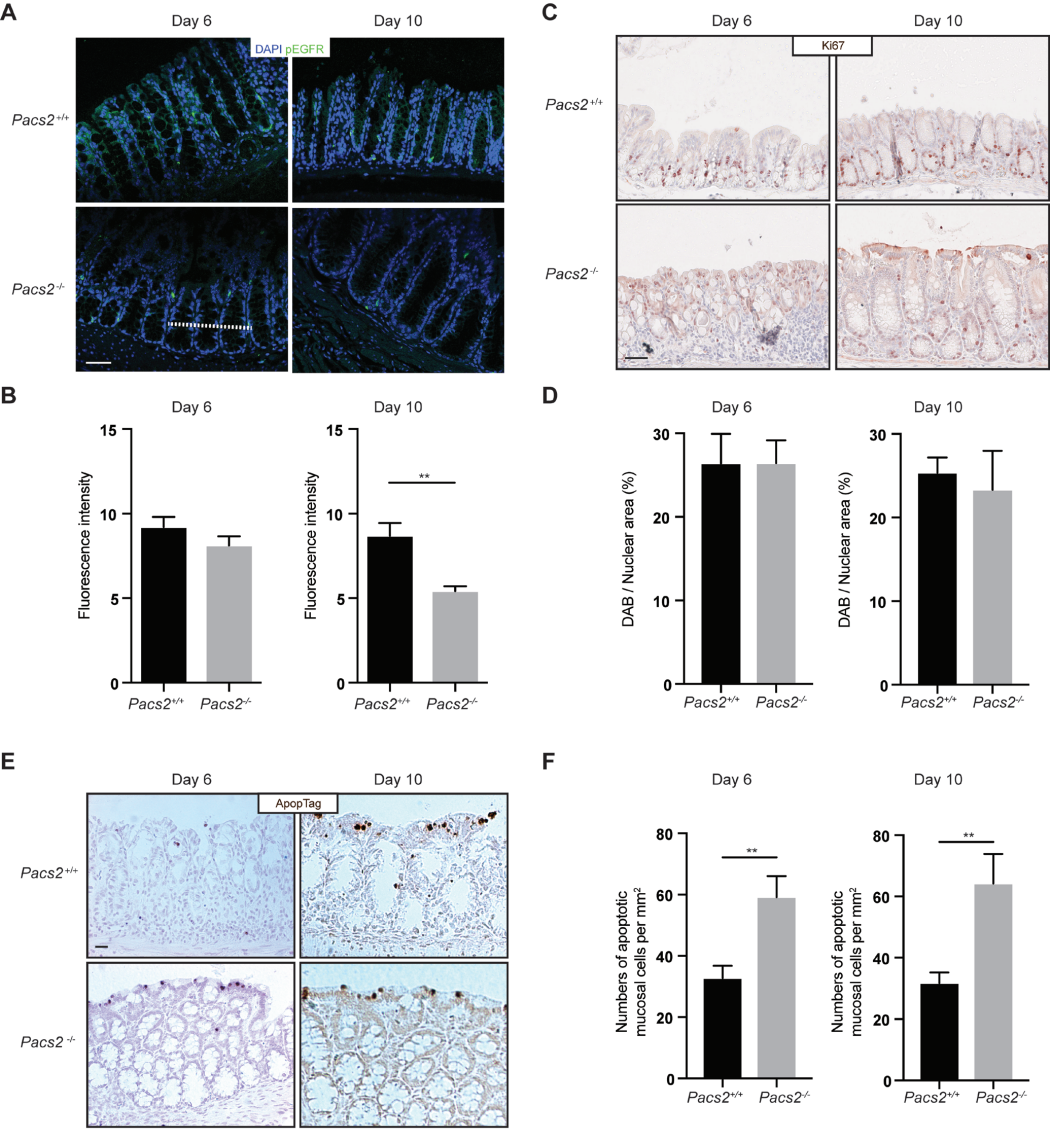
Evaluation of the regenerative response of intestinal epithelial cells in DSS-treated *Pacs2*^*-/-*^ mice **A.** Representative images of colonic specimens from control and *Pacs2*^-/-^ mice isolated at day 6 and day 10 and stained for phosphorylated EGFR (Tyr1068) and nuclear DAPI stain. **B.** The graph shows the pEGFR fluorescence intensity quantified along a line across colonic crypts (represented by the dotted line in A) on 5 equally positioned, random confocal images from each of 5 control and 5 *Pacs2-/-* mice. **C.** Representative images of colonic specimens from control and *Pacs2*^-/-^ mice isolated at day 6 and day 10 and stained for Ki67 positive proliferating cells. **D.** The graph shows Ki67 positive cells represented as the percentage diaminobenzidine (DAB) positive area per total hematoxylin stained nuclear area from 5 equally positioned, random images from each of 5 control and 5 *Pacs2-/-* mice. **E.** Representative images of colonic specimens from control and *Pacs2*^-/-^ mice isolated at day 6 and day 10 and stained for apoptotic cells by ApopTag. **F.** The graph shows the number of apoptotic cells per mm^2^ counted from 3 equally positioned, random images from each of 5 control and 5 *Pacs2-/-* mice. Scale bars = 50 µm. All graphs show mean values ± SEM analyzed by unpaired two-tailed Student’s t-test. **p < 0.01.

To examine the regenerative response of the intestinal epithelium to DSS treatment, we assessed the level of activated EGFR (Tyr1068 phosphorylated) by immunofluorescence staining of histological colon sections from *Pacs2*^*-/-*^ and control mice (Figure 3A and 3B). While no difference was observed at day 6 after DSS treatment, the level of activated EGFR was significantly reduced at day 10 of the DSS-induced colitis model in *Pacs2*^*-/-*^ mice compared to control mice. Yet, proliferation of the intestinal epithelial cells was not significantly affected at any of the time points, as assessed by Ki67 staining (Figure 3C and 3D). Since PACS-2 coordinates multiple cell survival pathways [2], we assessed the number of apoptotic cells after DSS treatment. In line with previous reports [21, 22], *Pacs2-/-* intestinal epithelial cells exhibited a marked increase in the number of apoptotic cell (Figure 3E and 3F).

For further evaluation of tissue damage and inflammation, mRNA levels of the inflammatory genes tumor necrosis factor (*Tnf)α*, interleukin *(Il)-6* and chemokine (C-X-C motif) ligand (*Cxcl)1*, as well as the EGFR ligands *Areg*, *Ereg* and *Tgfα* were analyzed by qRT-PCR. All transcripts, except *Tgfα* were upregulated on day 6 in the colons of both control and *Pacs2*^-/-^ DSS-treated mice, with no differences in expression of any of the genes between genotypes (Supplementary Figure 1A and 1B). Likewise, expression of the fibrotic marker collagen type 1 *(Col1)a1* was equally increased in *Pacs2*^-/-^ and control mice after DSS treatment (Supplementary Figure 1C). These findings were supported by ELISA data, demonstrating comparable AREG and TNFα levels in supernatants from tissue explant cultures established from mice sacrificed at day 6 of the DSS-induced colitis model (Supplementary Figure 1D).

### Pacs2-deficiency has no apparent effects on intestinal tumor growth in the *Apc*^*Min*^ mouse model of colorectal cancer

**Figure 4:**
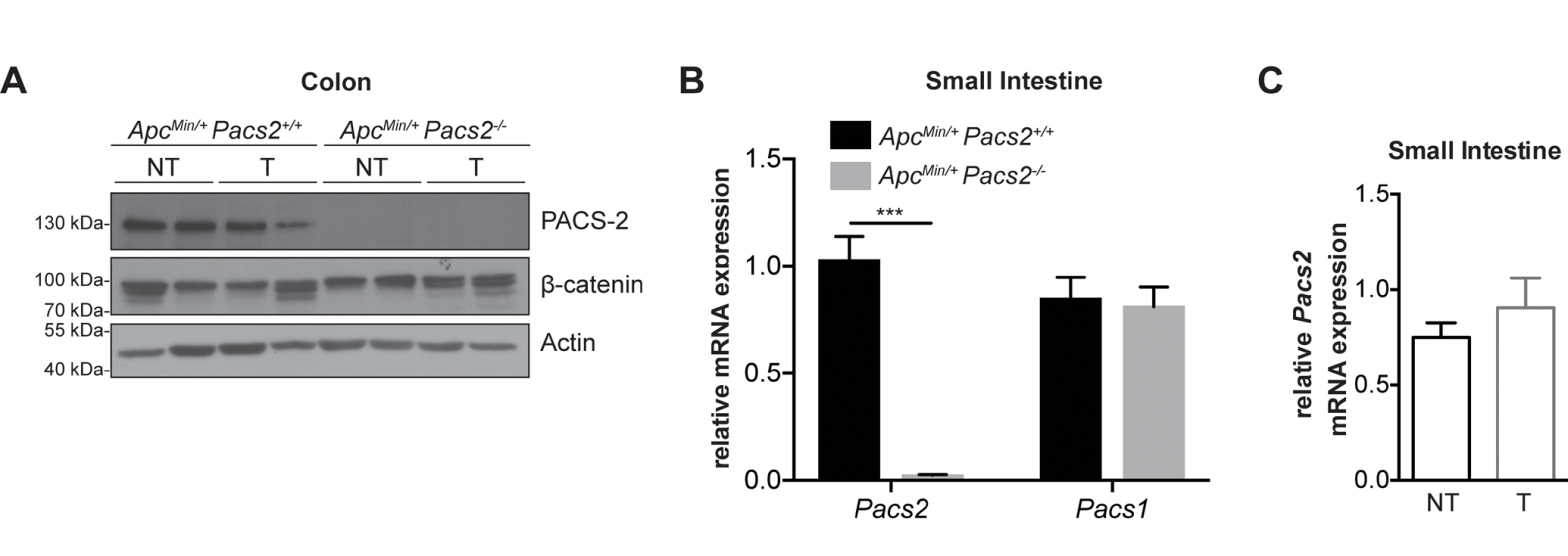
Characterization of *Apc*^*Min*/+^ *Pacs2*^*-/-*^ mice **A.** Representative cropped western blots showing expression of PACS-2 (top panel) and β-catenin (middle panel) in tumor (T) and non-tumor (NT) colonic tissue samples from littermate *Apc*^*Min/+*^ *Pacs2*^*+/+*^ (control) and *Apc*^*Min/+*^ *Pacs2*^*-/-*^ mice. Actin served as loading control. **B.** Quantitative real-time PCR analysis of *Pacs2* (left) and *Pacs1* (right) mRNA levels in the small intestine of control and *Apc*^*Min/+*^ *Pacs2*^*-/-*^ mice (control, *n* = 7; *Apc*^*Min/+*^ *Pacs2*^*-/-*^, *n* = 3). *Gapdh* served as a control for normalization. **C.** Quantitative real-time PCR analysis of *Pacs2* mRNA levels in tumor (T) and non-tumor (NT) tissue isolated from small intestine of *Apc*^*Min/+*^ *Pacs2*^*+/+*^ mice (NT, *n* = 5; T, *n* = 4). All data represent the mean ± S.E.M and were analyzed by unpaired Student’s t-test; ***p < 0.001.

Another process that relies on EGFR signaling is the development of intestinal tumors. We therefore evaluated the effects of *Pacs2*-deficiency in the *Apc*^Min^ mouse model of colorectal cancer. The successful generation of *Apc*^Min/+^ *Pacs2*^-/-^ mice was confirmed by western blot (Figure 4A) and qRT-PCR analysis (Figure 4B). PACS-2 was equally expressed in tumor and non-tumor tissues from the colons of *Apc*^Min/+^ *Pacs2*^+/+^ (control) mice as shown by western blot (Figure 4A), and confirmed by qRT-PCR analysis (Figure 4C). In contrast to our findings in the DSS-induced colitis model (Figure 1B), *Pacs2*-deficiency did not affect the small intestinal expression of PACS-1 (Figure 4B). Moreover, there were no differences in the overall colonic APC-regulated β-catenin expression levels between littermate control and *Apc*^Min/+^ *Pacs2*^-/-^ mice (Figure 4A).

**Figure 5:**
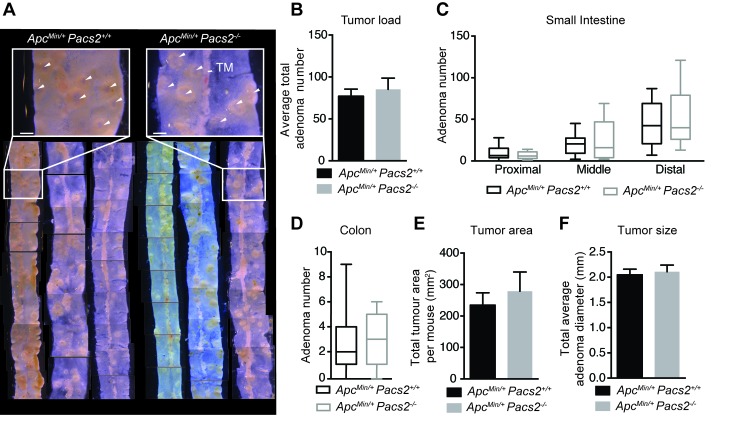
Pacs2-deficiency has no apparent effects on intestinal tumor growth in the *Apc*^*Min*^ mouse model of colorectal cancer **A.** Representative gross images of ileum specimens from control and *Apc*^Min/+^ *Pacs2*^-/-^ mice. White arrowheads indicate tumors. TM = tunica muscularis. Scale bar = 500 µm. Analysis of tumor number **B.**, tumor distribution in the proximal, middle, and distal small intestine **C.** and colon **D.**, tumor area **E.** and tumor size **F.** in control (*n* = 21) and *Apc*^Min/+^ *Pacs2*^-/-^ (*n* = 19) mice. All data represent the mean ± S.E.M (B, E, F) or the mean ± the minimum/maximum data points (C, D) and were analyzed by unpaired Student’s t-test.

Gross examination of isolated ileal tissue from control and *Apc*^Min/+^ *Pacs2*^-/-^ mice revealed no obvious differences in tumor load (Figure 5A). This was confirmed by quantification of the average total number of adenomas per mouse (Figure 5B). We found a similar distribution of tumors in *Apc*^Min/+^ *Pacs2*^-/-^ *versus* control mice, based on evaluation of the average number of adenomas in the proximal, middle and distal segments of the small intestine (Figure 5C) and entire colon (Figure 5D). Likewise, there were no changes in total tumor area per mouse (Figure 5E) or in tumor size, quantified as average adenoma diameter (Figure 5F).

### PACS-2 loss does not affect tumor morphology, β-catenin expression or cell proliferation in the *Apc*^*Min*^ model of colorectal cancer

**Figure 6:**
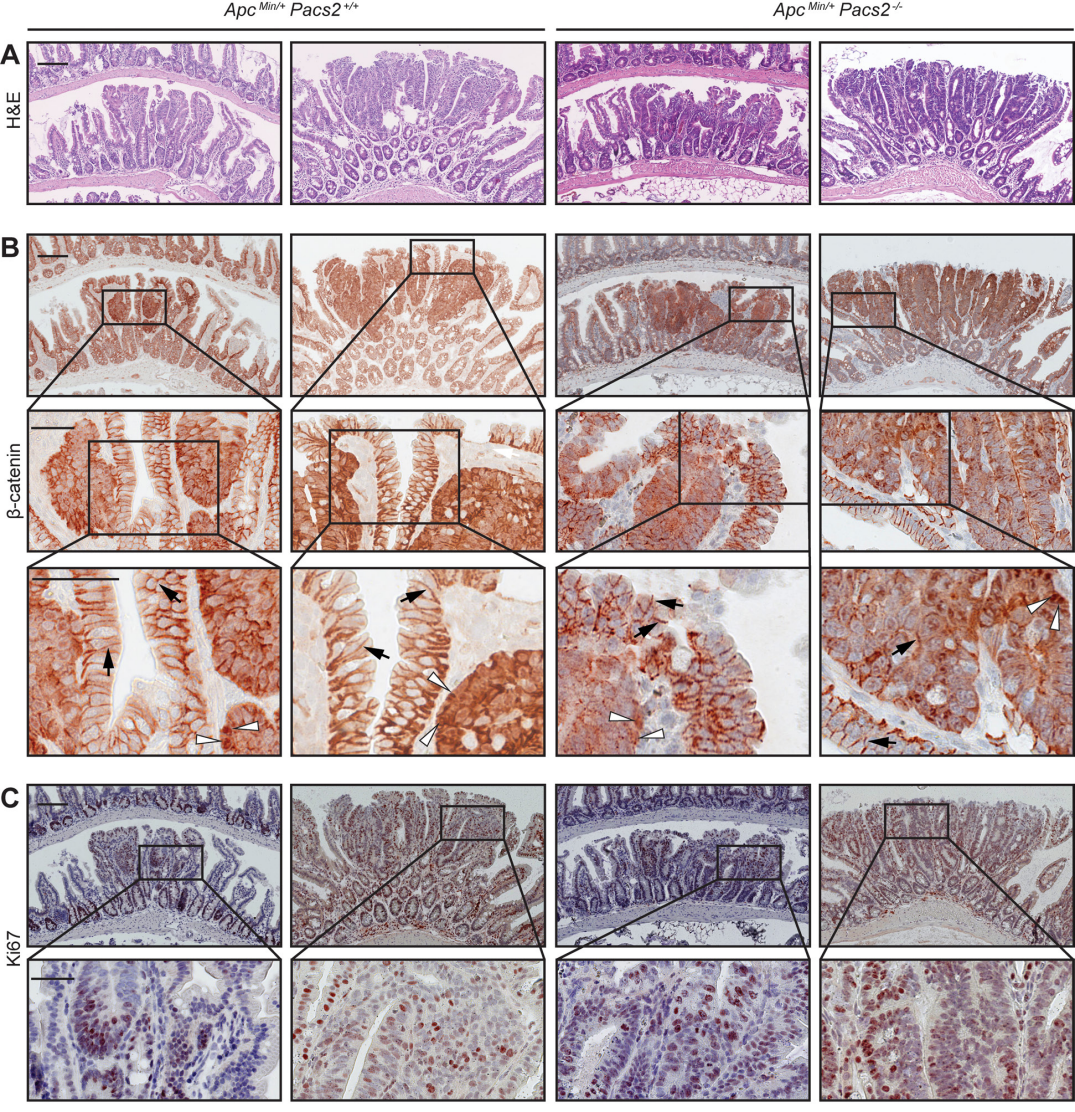
PACS-2 loss does not affect tumor morphology, ß-catenin expression or cell proliferation in the *Apc*^*Min*^ model of colorectal cancer Representative histological images of ileal adenomas from *Apc*^Min/+^ *Pacs2*^+/+^ (control) and *Apc*^Min/+^ *Pacs2*^-/-^ mice. **A.** depicts H&E staining (scale bars = 100 µm), **B.** immunohistochemistry for ß-catenin (upper panel 10x, scale bars = 100 µm; lower panels scale bars = 50 µm; white arrowhead = nuclear staining; black arrow = membrane staining), and **C.** shows staining for the proliferation marker Ki67 (upper panel 10x, scale bars = 100 µm; lower panel scale bars = 50 µm).

H&E staining of histological sections of adenomas in the distal small intestine revealed no obvious morphological changes between control and *Apc*^Min/+^ *Pacs2*^-/-^ mice (Figure 6A). To confirm constitutive Wnt signaling in tumors upon APC mutation, we performed immunohistochemistry for β-catenin. β-catenin was localized to the perinuclear region in tumors, but cell membrane-associated in the surrounding non-tumor tissue, with no apparent differences between control and *Apc*^Min/+^ *Pacs2*^-/-^ mice (Figure 6B). Also, Ki67 immunostaining showed comparable cell proliferation in both genotypes (Figure 6C).

## DISCUSSION

We recently reported that PACS-2 regulates cell-surface availability of ADAM17, thereby fine-tuning ADAM17 activity and downstream ErbB signaling [3]. We observed a marked reduction in phosphorylated EGFR levels and proliferative index in the small intestinal epithelium and to a lesser extent in the colonic epithelium of *Pacs2*^-/-^ mice. Furthermore, *Pacs2*^-/-^ mice had abnormal intestinal histology with a decrease in villus/crypt ratio of the small intestine [3]. Interestingly, intestinal abnormalities have been reported in a number of studies of genetic alterations in the ADAM17/EGFR axis [9, 23-28]. While these phenotypes are not directly comparable to those observed in *Pacs2*-deficient mice, we found the similarities interesting. Apart from maintaining normal intestinal homeostasis, ADAM17 and ErbB signaling is of great importance for intestinal epithelial regeneration, and in the development of intestinal tumors [7-11, 16, 28, 29]. Here, although we observed a slight effect of *Pacs2*-deficiency in the context of intestinal inflammatory damage, loss of the ADAM17 fine-tuning function of PACS-2 was not sufficient to influence tumor formation driven by excessive Wnt signaling in the *Apc*^Min^ model of colorectal cancer.

Several studies have demonstrated that *Adam17* and *Egfr*-deficient mice are more susceptible to colitis induced by oral administration of DSS. Typical findings include increased weight loss, higher mortality, and more severe inflammation and ulceration in these animals [8-11]. We observed a tendency towards poorer overall survival and slightly increased disease score in the *Pacs2*^-/-^ mice, but none of the changes were statistically significant. Also, while loss of ADAM17 has been shown to significantly impact both inflammation and regeneration in the DSS-induced model of colitis [8], PACS-2-deficient animals only displayed indications of delayed regeneration in this model. This modest impact of *Pacs2*-deficiency likely results from its fine-tuning effects on ADAM17, whereby loss of PACS-2 only decreases ADAM17 cell-surface levels and intestinal EGFR activation by approximately 50% [3]. Thus, PACS-2 loss cannot be expected to have as severe effects as loss of ADAM17 or EGFR. Indeed, although we did find significantly reduced pEGFR levels in the colon of *Pacs2-/-* mice as compared to control mice at day 10 of the DSS-induced colitis model, no significant difference in the number of proliferating colonic epithelial cells was observed. Corroborating these marginal effects, the previously reported reduction in EGFR phosphorylation and intestinal cell proliferation in PACS-2-deficient mice was much less pronounced in the colon as compared to the small intestine [3].

We observed no apparent effects of *Pacs2*-deficiency in the *Apc*^Min^ model of colorectal cancer when assessing tumor number, size or histology. While EGFR signaling is strongly implicated in the development of intestinal tumors [16], it appears that loss of the fine-tuning effects of PACS-2 is also not sufficient to impede tumor development or progression. It is possible that the strong proliferative signaling resulting from APC-deficiency overrides the modest anti-proliferative effects caused by the reduction in EGFR activity in *Pacs2*^-/-^ animals. Additionally, it has been shown that systemic overexpression of ADAM17 in mice does not lead to enhanced shedding activity or any overt abnormalities [30], and expression of only 5% of normal ADAM17 levels is enough to overcome the embryonic lethality associated with complete ADAM17 knockout in mice [8]. Hence, the amount of ADAM17 appears not to be rate-limiting, as long as it is above a certain threshold. It is therefore likely that the fine-tuning effects of PACS-2 serve to uphold a homeostatic balance in ADAM17 levels, and that the loss of this has little functional consequence when the inflammatory or proliferative signals are very potent.

Worth mentioning, we found a small but statistically significant increase in *Pacs1* expression in the colon of DSS-treated *Pacs2-/-* mice as compared to control mice. With a high degree of homology and frequently shared cargo of PACS proteins, this could indicate a potential functional compensation by PACS-1 upon loss of PACS-2 expression. Yet, no difference in PACS-1 mRNA levels were detected in the small intestine of control and *Apc*^Min/+^ *Pacs2*^-/-^ mice. Whether PACS-1 exerts similar regulatory effects on ADAM17-mediated EGFR activation as PACS-2 is a present unknown. For future studies, it would be interesting to investigate such potentially redundant functions as well as the phenotypic impact of deleting both genes.

Moreover, PACS-2 serves numerous other functions unrelated to ADAM17 and ErbB signaling. The loss of these other functions may serve to promote inflammation or tumor growth, and in this way neutralize any anti-inflammatory or anti-tumorigenic effects caused by reduced ADAM17 and ErbB signaling. Importantly, PACS-2 exerts highly complex regulatory functions on multiple cell death pathways (for a recent review see [2]). For example, PACS-2 is a key effector in TRAIL-mediated apoptosis, and loss of PACS-2 was shown to desensitize human colon cancer cells to TRAIL-induced apoptosis [31]. On the other hand, PACS-2 promotes cell survival following DNA damage, as exemplified by increased radiation-induced apoptosis in *Pacs2*^*-/-*^ thymocytes [21, 22]. In line with the later findings, we observed a significant increase in the number of apoptotic mucosal cells in the colon of DSS-treated *Pacs2*^*-/-*^ mice as compared to control mice.

Although the sample size was too small to determine the relative importance, it has been reported that the human *PACS2* locus is susceptible to loss of heterozygosity in colon cancer patients [32]. Also, a small selection of clinical material showed loss of PACS-2 in 4 out of 8 tumor samples, with PACS-2 expression being preserved in the surrounding non-tumor tissue [31]. While this finding needs validation in a larger patient cohort, a selective pressure for the loss of PACS-2 expression in tumor cells could indicate that the pro-apoptotic functions of PACS-2 may dominate effects on tumor formation and growth.

In conclusion, our data show that the effect of PACS-2 on ADAM17-mediated EGFR activation has negligible impact on DSS-induced colitis and the *Apc*^Min^ model of cancer. These observations suggest that as a fine-tuner of ADAM17 and ErbB activities, *Pacs2*-deficiency has no notable outcome on intestinal epithelial pathologies where the induction of inflammation or cell proliferation is sufficiently strong and the amount of ADAM17 not rate-limiting.

## MATERIALS AND METHODS

### Reagents and antibodies

DSS (Molecular weight 36,000-50,000 kDa) was obtained from MP Biomedicals (catalog no. 02160110). Hemoccult Test Kit was obtained from Beckman Coulter. Complete EDTA-free protease inhibitor cocktail was from Roche.

Primary antibodies used for immunohistochemistry (IHC) and western blotting (WB) were: mouse anti-actin (Millipore MAB1501R, diluted 1:1000 for WB), mouse anti-β-catenin (BD Transduction Laboratories #610154, diluted 1:1000 for IHC and 1:2000 for WB), rabbit anti-Ki67 (Abcam Ab15580, diluted 1:500 for IHC), rabbit anti-EGFR phosphorylated at Tyr1068 (Abcam Ab40815, diluted 1:200 for IHC). Rabbit anti-PACS-2 18193 was previously described [21], and was diluted 1:1000 for WB.

Enzyme-linked immunosorbent assay (ELISA) kits used for analysis of *ex vivo* colonic tissue culture supernatants were mouse amphiregulin DuoSet ELISA (R&D Systems DY989) and mouse TNF-α ELISA Ready-SET Go! (eBioscience 88-7324).

The primers used for *Pacs*2 genotyping were 5′-ATG CAT ACC TGC CCT TAG CAG AGG-3′, 5′-TGG AGT CTG AGG TTG AGG CCT TGA G-3′, and 5′-ATG GCG TTA CTT AAG CTA GCT TGC-3′. Primers for *Apc*^Min/+^ genotyping were 5′-GCC ATC CCT TCA CGTTAG-3′, 5′-TTC CAC TTT GGC ATA AGG C-3′ and 5′-TTC TGA GAA AGA CAG AAG TTA-3′. All primers were obtained from TAG Copenhagen.

### Animals

All animal studies were approved by the Danish Animal Inspectorate (License no. 2014-15-0201-00237). The authors confirm that all the experiments were performed in accordance with Danish guidelines and regulations for scientifically and ethically approved experimentation.

All mice were maintained in a climate-controlled room at a temperature of 22 ± 2°C and a relative humidity of 50 ± 5% under a 12-hour light/dark cycle and fed a standard diet. *Pacs2*^-/-^ and *Pacs2*^+/+^ littermate mice were co-housed and all handling of the mice, e.g. changing of cages, was done under non-sterile conditions to ensure similar microbiota in individual cages. *Pacs2*^-/-^ mice on the C57BL/6 background have been previously described [31] and were from The Jackson Laboratory. *Apc*^Min/+^ mice (C57BL/6 background) were acquired through The Jackson Laboratory. *Apc*^Min/+^ *Pacs*2^-/-^ mice were obtained by crossbreeding between *Apc*^Min/+^ mice and *Pacs2*^-/-^ mice. The founder mice were viable and exhibited normal growth. All genotyping was performed by polymerase chain reaction (PCR) using genomic DNA prepared from mouse-tails, using the primers listed above.

### DSS-induced mouse model of colitis

Littermate groups of male *Pacs2*^+/+^ (control) and *Pacs2*^-/-^ mice (8 to 16 weeks old) received 2.3% DSS (molecular weight 36,000-50,000 kDa, MP Biomedicals) in their drinking water for 5 days, followed by 5 days on regular drinking water. The optimal DSS concentration was carefully determined through several dose-response experiments starting with 2% DSS, which was the dose used in the study of hypomorphic *Adam17*^*ex/ex*^ mice [8]. The final experiment with 2.3% DSS was repeated three times, including a total of 21 control and 17 *Pacs2*^-/-^ mice. Mice were observed and weighed on days 0, 2, and 5-10. There was no significant difference in initial body weight between the two groups. Percentage weight was calculated as (weight on a given day / weight on day 0) x 100. Stool consistency and stool blood content were monitored on days 5-10 (see Figure 1B). Mice were sacrificed upon reaching the humane endpoints defined as more than 20% weight loss, severe pain or severe rectal bleeding. All surviving mice were euthanized on day 10 and entire colons were isolated and processed for histological analysis (see below).

Weight loss, stool consistency, and stool blood content were scored as described in Table 1, adapted from the method first described by Cooper et al. [12]. Stool blood content was first determined by visual inspection of stool. If no gross blood was visible, the presence of occult blood was determined using a Hemoccult Test Kit (Beckman Coulter). The DAI was calculated as the average of the three scores [12]. In the few cases where it was not possible to acquire a stool sample, the weight loss score was substituted for the DAI. To assess tissue damage, we employed a method adapted from that previously described [20]. Histological sections of entire colons were scored blindly and independently by two researchers, one of them a trained pathologist, and subsequently compiled. Epithelial damage and extent of inflammation were scored using the criteria outlined in Table 2. An overall histology score was then calculated as the sum of the epithelial damage score and the inflammation score [20].

### Analysis of intestinal tumors in the *Apc*^*Min*^ mouse model of colorectal cancer

Eighteen-week old littermate *Apc*^Min/+^ *Pacs2*^+/+^ (control, *n* = 22) and *Apc*^Min/+^ *Pacs2*^-/-^ (*n* = 19) mice (mixed sexes) were sacrificed, and the entire intestine was excised and divided into colon and three equal segments of small intestine: proximal, medial, and distal representing duodenum, jejunum and ileum, respectively. All regions were opened longitudinally and examined macroscopically for number, size and location of adenomas, using a stereomicroscope. Tissues were subsequently processed for histological analysis (see below).

### Histological analysis

Intestinal tissues were fixed in 4% paraformaldehyde (Sigma-Aldrich) dissolved in phosphate-buffered saline (PBS), embedded in paraffin, and sectioned to a thickness of 3.5 μm. H&E and IHC staining for β-catenin and Ki67 were performed as previously described [3]. The sections were scanned using a NanoZoomer 2.0-HT Digital slide scanner C9600 (Hamamatsu) and analyzed with NDP.view 2 software. Ki67 positive proliferating cells were quantified as the percentage diaminobenzidine (DAB) positive area per total hematoxylin stained nuclear area, using ImmunoRatio, a publicly available application for quantitative image analysis [33]. Activated EGFR levels were assessed as described previously [3], using an antibody against EGFR phosphorylated on Tyr1068 and Alexa Fluor 488 conjugated secondary anti-rabbit antibody, and measuring the fluorescence intensity along a line across colonic crypts. Apoptotic intestinal epithelial cells were detected using the ApopTag^®^ Peroxidase *In Situ* Oligo Ligation (ISOL) Apoptosis Detection Kit as recommended by the manufacturer (Chemicon), and quantified using Axiovision Software (Zeiss) and manual counting of stained cells per mm^2^ tissue from 3 randomly chosen mucosal areas. For quantifications, colon sections from 4-5 controls and 4-5 *Pacs2*^*-/-*^ mice were used.

### Western blotting

Intestines were opened longitudinally, and macroscopic tumors as well as non-tumor tissue was isolated. Protein extracts from non-tumor and tumor tissue were prepared using RIPA buffer (50 mM Tris pH 7.4; 150 mM NaCl; 0.1% SDS; 1% Triton X-100; 0.5% sodium deoxycholate; 1 mM EDTA, with protease inhibitor cocktail), and adjusted to equal concentration. Expression of PACS-2 and β-catenin were subsequently evaluated by western blot analysis using standard procedures, as described [3].

### Quantitative real-time PCR

Colonic tissue or intestinal macroscopic tumors and corresponding non-tumor tissue were isolated from intestines. To stabilize RNA, tissue was immersed in RNA*later* solution (Sigma-Aldrich) and then snap-frozen in liquid nitrogen. Total RNA was extracted from tissues using the GeneJET RNA Purification Kit (ThermoFisher Scientific) according to the manufacturer´s instructions. Total RNA (1-2 µg) was reverse transcribed using RevertAid Reverse Transcriptase (ThermoFisher Scientific, cat. no. EP0441), and quantitative real-time PCR (qRT-PCR) was performed on a LightCycler480 PCR machine (Roche) using Maxima SYBR Green/ROX qPCR Master Mix (2X) (ThermoFisher Scientific, cat. no. K0221), according to the manufacturer’s instructions. The following combinations of primers were used to analyze the expression of *Pacs2*: 5´-CCT GGC TCT GAC CTT CTC TC-3´ and 5´-CCA GGA TCG TCC TGT TCT TG-3´. *Pacs1* primers were: 5´-CAG CAC CTT TCT TGA TTC TGC-3´ and 5´-TCC ATT GAC ATA CTG CAT CAC A-3´. Primers for *gapdh* were: 5´-AAG GTC ATC CCA GAG CTG AA-3´and 5´-CTG CTT CAC CAC CTT CTT GA-3´. *Areg* primers were: 5´-TCC AAG ATT GCA GTA GTA GCT GTC-3´ and 5´- CCC TGA AGT ATC GTT TCC AAA G-3´. *Ereg* primers were: 5´-CAC CGA GAA AGA AGG ATG GA-3´and 5´-TCA CGG TTG TGC TGA TAA CTG-3´. *Tgfα* primers were: 5´-CCT GGT GGT GGT CTC CAT T-3´ and 5´-CAG TGT TTG CGG AGC TGA-3´. *Tnfα* primers were: 5´-CTG TAG CCC ACG TCG TAG C-3´and 5´-TTG AGA TCC ATG CCG TTG-3´. *Il-6* primers were: 5´-GCT ACC AAA CTG GAT ATA ATC AGG A-3´and 5´-CCA GGT AGC TAT GGT ACT CCA GAA-3´. *Cxcl1* primers were: 5´-GAC TCC AGC CAC ACT CCA AC-3´and 5´-TGA CAG CGC AGC TCA TTG-3´. *Col1a1* primers were 5´-CTG ACT GGA AGA GCG GAG AGT-3´ and 5´-GAC GGC TGA GTA GGG AAC AC-3´. Data were analyzed using the 2−∆∆CT methodology, as described [34].

### *Ex vivo* cultures of colonic explants

Tissue explants sized approximately 1 cm were harvested from the proximal colon and washed twice in cold PBS containing 100 µg/ml of streptomycin and 100 U/ml penicillin (with incubation for at least 10 min). After washing the tissue specimens, cultures were placed in a 24-well plate in 1 ml of DMEM with 100 µg/ml of streptomycin and 100 U/ml penicillin. After 24 hours, supernatants were collected, centrifuged at 1000 x g to eliminate debris, and analyzed by ELISA.

### ELISA

Soluble TNFα and AREG levels were measured by ELISA in cell-free *ex vivo* colonic tissue culture supernatants according to the manufacturer´s instructions. Absolute values were normalized to tissue weight of colonic culture explants used in the experiment.

### Statistical analysis

Unless otherwise stated, all data represent the mean ± standard error of the mean (S.E.M.). Data were analyzed by unpaired Student’s t-test, analysis of variance (ANOVA) with Tukey’s post-test, or log-rank (Mantel-Cox) test, as appropriate, using GraphPad Prism version 6.00 for Macintosh (GraphPad Software). In all cases, p < 0.05 was considered statistically significant.

## SUPPLEMENTARY MATERIALS FIGURE



## Abbreviations

(ADAM17): A disintegrin and metalloproteinase-17
(APC): Adenomatous polyposis coli
(AREG): Amphiregulin
(ANOVA): Analysis of variance
(Cxcl)1: Chemokine (C-X-C motif) ligand
(DSS): Dextran sodium sulfate
(DAI): Disease Activity Index
(ELISA): Enzyme-linked immunosorbent assay
(EGFR): Epidermal growth factor receptor
(EREG): Epiregulin
(H&E): Hematoxylin and eosin
(IHC): Immunohistochemistry
(PACS-2): Interleukin (Il)-6, Phosphofurin acidic cluster sorting protein 2
(qRT-PCR): Quantitative real-time PCR
(S.E.M.): Standard error of the mean
(TGF-α): Transforming growth factor
(TNF-α): Tumor necrosis factor
(WB): Western blotting
