# Supplementary material for: Loss of PACS-2 delays regeneration in DSS-induced colitis but does not affect the ApcMin model of colorectal cancer
Source: Oncotarget. 2017 Nov 26;8(65):108303–15. doi: 10.18632/oncotarget.22661 (PMC5752446; doi:10.18632/oncotarget.22661)
Supplement: Supplementary file 1 [file oncotarget-08-108303-s001.pdf]

Loss of PACS-2 delays regeneration in DSS-induced colitis but does not affect the *Apc*^{Min} model of colorectal cancer

SUPPLEMENTARY MATERIALS

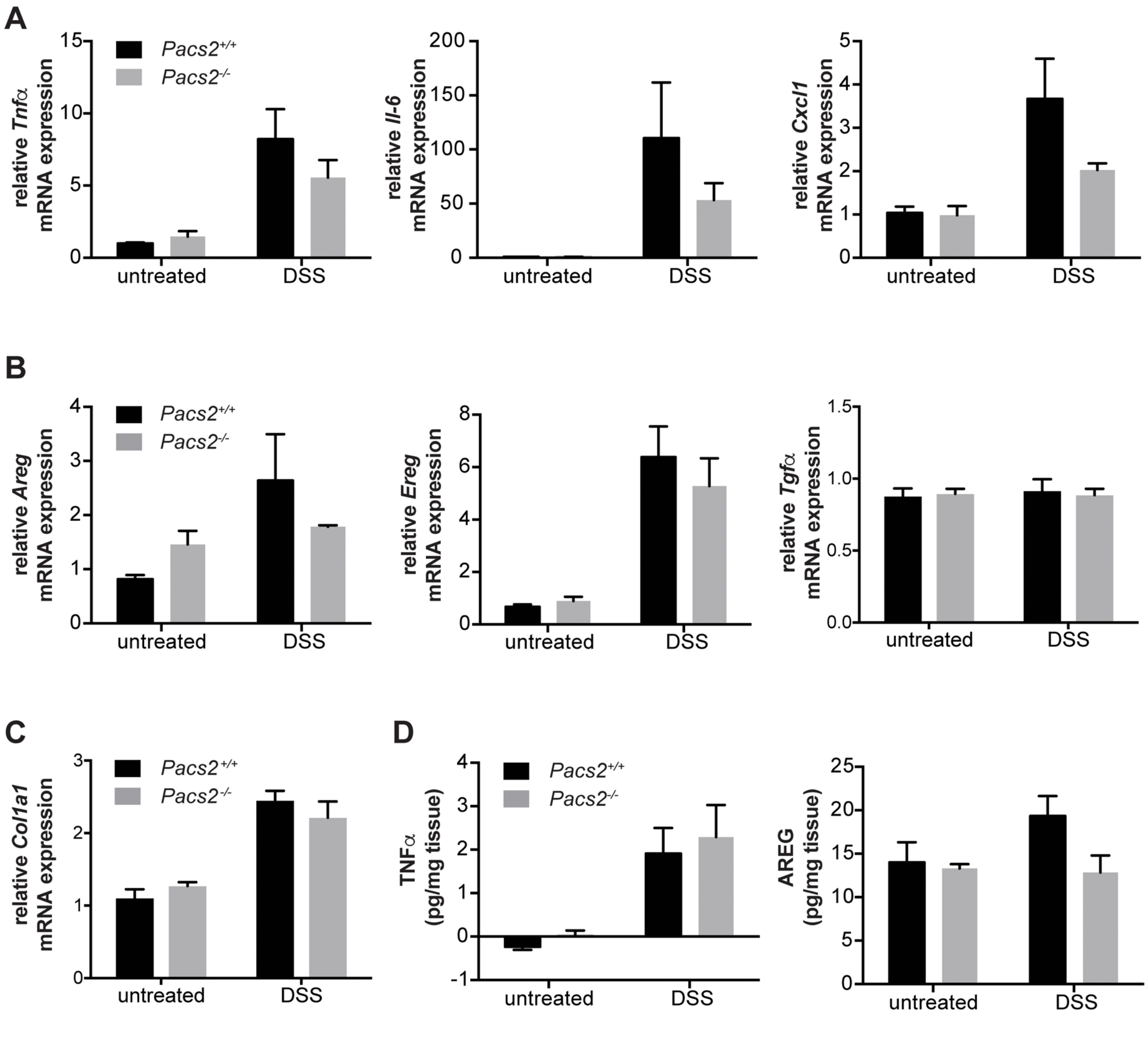

Supplementary Figure 1: Evaluation of inflammatory gene expression in DSS-treated Pacs2$^{-/-}$ mice. Quantitative real-time PCR analysis of (**A**) inflammatory genes *Tnfα, Il6 and Cxcl1,* (**B**) EGFR ligands *Areg, Ereg and Tgfα*, or (**C**) the fibrosis marker *Col1a1*. mRNA was isolated from colonic tissue of DSS-treated control and *Pacs2*$^{-/-}$ mice on day 6 of the DSS protocol (control, n=5; *Pacs2*$^{-/-}$, n=4). (**D**) ELISA of AREG and TNFα levels in supernatants from tissue explant cultures established at day 6 of DSS treatment (control, n=5; *Pacs2*$^{-/-}$, n=4). All data represent the mean ± S.E.M and were analyzed by two-way ANOVA including Tukey's post-test.
